# Prevalence of Emergency Department Social Risk and Social Needs

**DOI:** 10.5811/westjem.2020.7.47796

**Published:** 2020-10-06

**Authors:** Melanie F. Molina, Caitlin N. Li, Emily C. Manchanda, Benjamin White, Mohammad K. Faridi, Janice A. Espinola, Henry Ashworth, Gia Ciccolo, Carlos A. Camargo, Margaret Samuels-Kalow

**Affiliations:** * Massachusetts General Hospital, Department of Emergency Medicine, Boston, Massachusetts; † Boston Children’s Hospital, Division of Emergency Medicine, Boston, Massachusetts; ‡ Boston Medical Center, Department of Emergency Medicine, Boston, Massachusetts; § Harvard Medical School, Boston, Massachusetts

## Abstract

**Introduction:**

Social risks, or adverse social conditions associated with poor health, are prevalent in emergency department (ED) patients, but little is known about how the prevalence of social risk compares to a patient’s reported social need, which incorporates patient preference for intervention. The goal of this study was to describe the relationship between social risk and social need, and identify factors associated with differential responses to social risk and social need questions.

**Methods:**

We conducted a cross-sectional study with 48 hours of time-shift sampling in a large urban ED. Consenting patients completed a demographic questionnaire and assessments of social risk and social need. We applied descriptive statistics to the prevalence of social risk and social need, and multivariable logistic regression to assess factors associated with social risk, social need, or both.

**Results:**

Of the 269 participants, 100 (37%) reported social risk, 83 (31%) reported social need, and 169 (63%) reported neither social risk nor social need. Although social risk and social need were significantly associated (p < 0.01), they incompletely overlapped. Over 50% in each category screened positive in more than one domain (eg, housing instability, food insecurity). In multivariable models, those with higher education (adjusted odds ratio [aOR] 0.44 [95% confidence interval {CI}, 0.24–0.80]) and private insurance (aOR 0.50 [95% CI, 0.29–0.88]) were less likely to report social risk compared to those with lower education and state/public insurance, respectively. Spanish-speakers (aOR 4.07 [95% CI, 1.17–14.10]) and non-Hispanic Black patients (aOR 5.00 [95% CI, 1.91–13.12]) were more likely to report social need, while those with private insurance were less likely to report social need (private vs state/public: aOR 0.13 [95% CI, 0.07–0.26]).

**Conclusion:**

Approximately one-third of patients in a large, urban ED screened positive for at least one social risk or social need, with over half in each category reporting risk/need across multiple domains. Different demographic variables were associated with social risk vs social need, suggesting that individuals with social risks differ from those with social needs, and that screening programs should consider including both assessments.

## INTRODUCTION

Social determinants of health (SDoH) affect health outcomes and healthcare utilization.^1^ The World Health Organization defines SDoH as “conditions in which people are born, grow, live, work and age,” which are “shaped by the distribution of money, power and resources at global, national and local levels.”^2^ These conditions include housing, income, education, transportation systems, neighborhoods, and many others. In a recent study evaluating the association between income and life expectancy, there was a 10- to 15-year difference between the richest 1% and the poorest 1%.^3^ Additionally, housing instability and food insecurity have been associated with increased emergency department (ED) use and hospitalizations.^4^ With rising pressures to improve health outcomes, reduce healthcare costs, and the transition from fee-for-service to accountable care organizations, the US healthcare system has become increasingly focused on identifying and addressing patients’ SDoH.^5^ Although most screening efforts have primarily focused on the outpatient clinical setting,^6^,^7^ studies have shown an association between adverse SDoH and ED visits.^8^,^9^ This relationship suggests that encounters in the ED may provide a unique screening opportunity, as many individuals who use the ED for healthcare may not otherwise have access to outpatient services, 8–10 and the ED may be their only opportunity for screening and intervention.

While SDoH may affect health for better or worse, *social risk* is defined as “specific adverse social conditions that are associated with poor health, like social isolation or housing instability.”^11^ Recently, Alderwick et al proposed a distinction between social risk and social need in order to incorporate patients’ preferences and priorities.^11^ In contrast to social risk, *social need* refers to the patient’s perceptions of adverse SDoH for which they would like assistance, allowing for patient prioritization of social interventions.^11^ Although subtle, this distinction is paramount, as there may be important differences between positive answers to screening questions about social risk vs social need, which in turn have critical implications for targeting interventions. For example, one study investigated screening for food insecurity using a screening questionnaire (social risk) vs a referral menu, the latter of which offered assistance obtaining food (social need).^12^ While the authors found that 31% reported food insecurity and 32% desired referrals to food resources, only 17% reported both.^12^ This implies that those who have social risk factors (ie, those who screen positive on a questionnaire inquiring about food insecurity) may not necessarily perceive themselves as needing extra resources (assistance with obtaining food).

The incomplete overlap highlights the importance of screening separately for social risk and social need, as the incorporation of patient preference for social assistance (ie, the expression of social need) is fundamental to understanding how and when to best connect patients to resources. Furthermore, it is unclear whether the same populations of patients who are screening positive for social risk are also screening positive for social need, and there are limited studies comparing patient answers to those questions across multiple domains. Thus, understanding the similarities and differences between social risk and social need screening with a multi-domain standardized questionnaire is important to determine which patients will most benefit from social interventions and how best to design those interventions.

**Population Health Research Capsule**

What do we already know about this issue?

*Social risk refers to adverse social conditions associated with poor health; social need refers to adverse social conditions with which patients would like assistance*.

What was the research question?

What was the prevalence of social risk and social need among ED patients, and how were they related?

What was the major finding of the study?

*Social risk/need were present in 1/3 of patients and significantly associated, but with incomplete overlap*.

How does this improve population health?

*Understanding the relationship between social risk and social need will improve screening for adverse social determinants of health that can subsequently be addressed*.

Existing screening tools have primarily focused on social risk alone and have used a heterogeneous set of questions.^6^,^8^,^13^ In an attempt to standardize screening, the Centers for Medicare & Medicaid Services (CMS) and National Academy of Medicine recently published a screening tool focusing on social risk in five domains: housing instability; food insecurity; transportation needs; utility needs; and interpersonal safety.^14^ However, the length of the CMS tool makes it challenging to use in time-limited settings such as the ED, and some of the questions remain under copyright protection. Furthermore, the CMS tool assesses social risk, but does not assess social need.

The objectives of this study were to describe and identify the following: 1) the prevalence of social risk and social need among patients in a large, urban ED using a brief screening tool; 2) the relationship between positive screens for social risk and social need; and 3) patient factors associated with differential responses to social risk and social need questions.

## METHODS

### Study Design

We conducted a cross-sectional study with 48 hours of time-shift sampling (spanning all 24 weekday hours and 24 weekend hours, 12 am-11:59 pm) between September 2018–April 2019 in each of five treatment areas within a large, urban, academic ED, with a yearly patient census of 114,433 (2019). The sampling method was designed to eliminate sampling bias associated with the inherently different patient populations likely to report to the ED during different times (weekday vs weekend or daytime vs nighttime) as well as with differing levels of acuity (ie, in a fast track vs higher acuity area of the ED). Bilingual (English-Spanish) research assistants (RA) approached patients for eligibility, and consenting patients completed both a brief demographic questionnaire and the social risk/need assessment. The assessment consisted of two sections, one assessing social risk and another assessing social needs, in each of the five recommended domains outlined by the National Academy of Medicine^14^ for standardized screening (Table 1).

Given that the CMS tool was under copyright restriction, we adapted the tool, using similar, publicly available and previously reported social risk questions in each domain. With regard to social need, given there is no existing validated screening tool spanning multiple domains, we added explicit, simplified questions regarding patient desire for social assistance across the same five domains. This method is similar to that employed in other studies assessing social need.^12^ Notably, others have highlighted the lack of gold standards for SDoH screening tools,^15^ the limited data on psychometric properties of screening tools,^16^ the large variation in prevalence of SDoH across domains, and the variable availability of community services across geographic locations that limits those SDoH that may be amenable to intervention.^15^ Given that these limitations preclude a formal validation of the tool, we felt that using questions from the scientific literature was the next best option.

In a private room, the RA verbally administered the survey to the participant, recording all responses directly into the secure online REDCap system. Patients were asked first about social risk and then social need. The survey altogether took approximately 5–7 minutes. Of note, regardless of screening results, all participants were provided with a sheet of local resources mapping to the domains of the survey. The study was approved by the institutional review board of Partners HealthCare.

### Selection of Participants

During each sampling shift in the ED, all newly arriving eligible patients and parents of pediatric patients (<18 years of age) entering the treatment area who spoke English or Spanish were approached for participation. Exclusion criteria included determination by the attending physician that the patients were inappropriate for enrollment, eg, intoxication or altered mental status to the degree of inhibiting decision-making capacity, or high medical acuity requiring immediate attention (such as emergent intubation or active resuscitation).

### Outcomes

The primary outcome was the prevalence of social risk and social need in a large, urban, academic ED. Secondary outcomes included the association between social risk and social need, as well as the association of demographic variables with social risk and social need, respectively.

### Analysis

We used descriptive statistics to summarize participants’ demographic characteristics and the prevalence of social risk and social need. We employed multivariable logistic regression models to assess the association between social risk and social need with demographic characteristics, including gender, race/ethnicity, language, education, health literacy, and insurance. For the multivariable logistic regression models, education was divided into two groups—high school or less vs some college or more—given the small number of participants with less than eighth-grade education. This cutoff is further supported by studies showing significant association of comprehension^17^ and mortality^18^ among those who have graduated high school and attained some college compared to those who have not. Given the potential colinearity between education and health literacy, these two variables were analyzed in two different models. We conducted analyses in STATA 15 (StataCorp, College Station, TX).

## RESULTS

### Characteristics of Study Subjects

Of the 614 patients or parents of patients who were approached, 483 (79%) were eligible for participation, with the primary reasons for ineligibility being intoxication and high medical acuity. Of the 483 eligible patients, 269 (56%) patients consented to and completed the survey. Eligible patients who did not participate did so because they were either transported elsewhere for a diagnostic procedure (eg, imaging) or declined participation, citing disinterest or pain. Among the 269 participants, 79 (29%) had completed only an elementary or high school education, and 121 (45%) had public or no health insurance. Twenty-four participants (9%) chose to complete the survey in Spanish.

### Main Results

Overall, 100 participants (37%) screened positive for social risk, while 83 (31%) screened positive for social need. Regarding social risk questions by domain, 23% were positive for housing insecurity, 17% for food insecurity, 9% for transportation needs, 4% for utility needs, and 17% for neighborhood safety concerns. Regarding social need, 15% screened positive for housing insecurity, 13% for food insecurity, 11% for transportation needs, 17% for utility needs, and 11% for safety concerns. Results for the individual questions are shown in Table 2. Of those 100 individuals who reported social risk, 57 (57%) reported having more than one social risk, and 45 of 83 (54%) reported more than one social need—suggesting a high co-prevalence across multiple domains. There was a significant association, but incomplete overlap, between the presence of social risk and social need in each domain (Table 3).

In unadjusted analyses, education was significantly associated with social risk and social need, with those patients having lower education being more likely to report the presence of both. Language, race/ethnicity, and insurance were also associated with social need but not social risk (Table 4); those patients who were Spanish-speaking, non-Hispanic Black, and/or possessed state/public insurance were more likely to report social need.

We created two multivariable logistic regression models, one for social risk and one for social need. Models 1A and 2A controlled for gender, race/ethnicity, language, education, and insurance status. Models 1B and 2B controlled for the same variables, with the exception of education, which was exchanged for health literacy. With regard to social risk, Model 1A demonstrated that participants who possessed higher than high school education had lower odds of reporting social risk (adjusted odds ratio [aOR] 0.44 [95% confidence interval [CI], 0.24–0.80]). Model 1B demonstrated that participants with private insurance had lower odds of reporting social risk (aOR 0.50 [95% CI, 0.29–0.88]) (Table 5). With regard to social need, Model 2A demonstrated that the characteristics independently associated with higher odds of reporting social need were Spanish speakers (aOR 4.07 [95% CI, 1.17–14.10]) and non-Hispanic Black race (aOR 5.00 [95% CI,1.91–13.12]). These results were corroborated by Model 2B: Spanish speakers (aOR 3.57 [95% CI, 1.01–12.57]) and non-Hispanic Black patients (aOR 4.96 [95% CI, 1.88–13.11]) (Table 6). Additionally, in both models, private, self-pay/none and unknown insurances were all associated with lower odds of reporting social need than those with state/public insurance, suggesting that those with state/public insurance were more likely to report social need.

## DISCUSSION

In a sample of 269 patients in a large, urban, academic ED, we found a high prevalence of social risk (37%) and social need (31%), with over 50% of those who reported either social risk or social need screening positive in more than one domain. Additionally, although answers to social risk and social need questions were significantly associated among all domains, the overlap was incomplete. This study employed an adaptation of a standardized screening tool spanning the five domains proposed by CMS^14^ to screen for social risk, with the addition of social need questions. Prior studies have either focused on one social risk or need^12^ or have identified a heterogeneous set of social risks or social needs specific to their study populations.^7^,^13^,^19^

Attempts to address these SDoH have included the creation of an ED-based help desk staffed by volunteers to help with patient navigation,^13^ the development of coordinated care models,^20^ partnership with community resources,^21^ and intervention programs targeting specific SDoH, such as interpersonal safety.^22^ However, understanding the co-prevalence of social risk and social need across multiple domains is important, particularly when designing interventions, as social needs in one domain may directly affect those in other domains. An intervention that targets social need in one domain without considering the patient’s needs across other domains may prove ineffective. For example, a program that addresses food insecurity by providing canned foods requiring reheating would be of limited benefit to a homeless individual (one with housing instability) who has no means to easily store or cook the food. Thus, screening across multiple domains provides a more comprehensive picture of an individual’s needs, such that each need can be identified and addressed with appropriate interventions. The optimal resource-linkage strategies are less clear and outside the scope of this paper; however, ideally they would be comprehensive and brief to ensure scalability.

This study also enabled the multi-domain direct comparison of social risk vs social need with two separate sets of questions. A prior study in pediatric outpatient clinics found limited overlap between screening positive for food insecurity and desiring referrals to food resources.^12^ Our study extends these results to adult and pediatric patients in the ED—screening individuals who may not otherwise have access to outpatient services—demonstrating incomplete overlap across multiple domains. The implications of this incomplete overlap are important to consider in designing interventions to improve a patient’s SDoH. By way of illustration, it may be that an individual who frequently has an insecure food supply is adequately connected to existing resources and does not need further support at the present time (social risk without social need). Similarly, another individual may in the short term have a stable housing situation, while simultaneously knowing that a future event (eg, rent increase at lease renewal) will lead to a more precarious position; they may thus need additional housing resources (social need without social risk).

Furthermore, this study exposed notable differences among patient factors associated with screening results for social risk vs social need. For example, language, race/ethnicity, and insurance status were significantly associated with social need, but not social risk. These results have several implications. First, directly soliciting social needs as opposed to social risk may be more sensitive for particular populations. Different groups may be more or less comfortable asking for or accepting support. Thus, programs focused only on social risk screening may undercount the social needs of their patient population and subsequently miss important opportunities for intervention. Second, given the time constraints of the ED, it may be preferable to screen for social need over social risk, given that doing so inherently allows patients to express their priorities. The utility of social risk screening may be primarily in predicting patients’ future healthcare utilization^8^,^9^ and understanding underlying population-level risk, rather than identifying individual patients who would be willing to receive social assistance.

Additionally, the significant association of language, race/ethnicity, education, and insurance status with the presence of social needs emphasizes the importance of screening in multiple languages, with program and referral materials that are accessible to patients across a broad range of educational attainment and health literacy. Furthermore, the high rate of co-prevalence of social risk and social need across domains suggests that screening should target multiple domains, in addition to assessing both social risk and social need. In our study, the brevity of the screening process allowed it to be accomplished during the ED visit without significant disruption in care—suggesting it may be performed at time of registration or in the waiting room, with few additional resources required. To minimize the personnel required for screening, electronic screening may be considered for future studies.

## LIMITATIONS

Our study has several limitations. First, the sample size was relatively small, which could lead to the under-detection of social risk and social need, as well as their associated demographic variables. Additionally, although the sampling strategy was carefully balanced across days of the week and times of day, the study captured 269 (56%) patients who were eligible to participate, leaving a significant proportion of patients – 214 (44%) – who were eligible but were unable or did not consent to being screened. Although this raises the potential for sampling bias, it also likely represents the “real-life” population of patients who would be screened in the ED, as patients who are disinterested, in significant pain, or undergoing necessary diagnostic studies would also be unlikely to respond to screening by their ED providers. Nevertheless, for future studies there may be opportunities to increase enrollment by providing incentives to participate, or enrolling patients later in their clinical course. Such studies would clarify the impact of non-participation—both in research and, presumably, future clinical screening—on the observed prevalence of social risk and social need.

Future studies might also consider temporality and its effects on social risk and social need, ie, patients presenting at the beginning of the month may have different needs than those presenting at the end of the month. Similarly, patients presenting during the summer months may have different needs than those presenting during the winter months. One study illustrating the former concept demonstrated that low-income individuals were more likely to report to the ED for hypoglycemia at the end of the month, as opposed to the beginning of the month.^23^

With regard to external validity, this study recruited participants from a large, urban, academic ED in the US. The prevalence of social risk and social need was thus specific to this population. The generalizability to hospitals serving different (eg, more rural, racially diverse, or socioeconomically disadvantaged) populations is limited. However, studies suggest that social risk and social need are widely prevalent in EDs across the country.^9^,^24^,^25^,^26^

Lastly, the topics broached in the patient interviews related to social risk and social need are considered sensitive and are often kept private. As a result, participants may not always disclose accurate information, which may lead to the under-detection of social risk/need. Ultimately, however, the determination of social risk and social need is dependent on self-report, as there is no gold standard for assessing true prevalence.^12^ Furthermore, in this study we asked first about social risk and then social need. To our knowledge, whether the order in which these questions are asked affects patient response is not known and merits further study.

## CONCLUSION

In summary, these data demonstrate that multi-domain, as opposed to single-domain, screening is necessary, given the high rate of co-prevalence of social risk and social need. Although there is significant overlap among those who screen positive for social risk vs social needs, there remain notable differences that merit further consideration when optimizing screening tools and designing interventions. These data also suggest that strategies aiming to identify and address social risk and social need should be accessible and easy to understand for those with limited education or health literacy. Future research questions include how best to conduct screening within the ED (eg, in-person vs electronic), how to successfully connect patients to social services, and whether these linkage strategies should be employed during the ED visit or after discharge.

## Figures and Tables

**Table 1:** Social risk and social need questions.

Domain	Questions	Sources
Social risk		

Housing instability	1a. In the last month, have you slept outside, in a shelter or in a place not meant for sleeping? 1b. In the last month, have you had concerns about the condition or quality of your housing? 1c. In the last 12 months, how many times have you or your family moved from one home to another? 1d. Are you worried that in the next 2 months, you may not have stable housing?	HealthBegins^27^ Health Leads^28^
Food insecurity	2a. Within the past 12 months, we worried whether our food would run out before we got money to buy more. 2b. Within the past 12 months, the food we bought just didn’t last and we didn’t have money to get more.	American Academy of Pediatrics^29^
Transportation needs	3a. How often is it difficult to get transportation to or from your medical or follow-up appointments? 3b. How often is it difficult to get transportation to or from your other non-medical activities (work, school etc.)?	HealthBegins^27^*
Utility needs	4. In the past 12 months, have you had any utility (electric, gas, water or oil) shut off for not paying your bills?	Health Leads^28^*
Interpersonal safety	5a. Do you have any concerns about safety in your neighborhood? 5b. Are you afraid you might be hurt in your apartment building or house?	HealthBegins^27^ Health Leads^28^

Social need†		

Housing instability	Would you like help with shelter or housing?	
Food insecurity	Would you like help with obtaining food?	
Transportation needs	Would you like help with transportation?	
Utility needs	Would you like help paying for your utility bills?	
Interpersonal safety	Would you like help regarding your personal or neighborhood safety?	

* Question has been slightly modified for ease of understanding in the ED setting.

† Questions internally developed.

**Table 2:** Prevalence of social risk and social need, by question and by group, N = 269.

Questions	n	%
Social risk		

In the last month, have you slept outside, in a shelter or in a place not meant for sleeping?	18	7
In the last month, have you had concerns about the condition or quality of your housing?	35	13
In the last 12 months, how many times have you or your family moved from one home to another?	16	6
Are you worried that in the next 2 months, you may not have stable housing?	37	14
Housing total	61	23
Within the past 12 months, we worried whether our food would run out before we got money to buy more.	35	13
Within the past 12 months, the food we bought just didn’t last and we didn’t have money to get more.	34	13
Food total	45	17
How often is it difficult to get transportation to or from your medical or follow-up appointments?*	20	7
How often is it difficult to get transportation to or from your other non-medical activities (work, school, etc.)?*	19	7
Transportation total	24	9
In the past 12 months, have you had any utility (electric, gas, water or oil) shut off for not paying your bills?	11	4
Utility total	11	4
Do you have any concerns about safety in your neighborhood?	40	15
Are you afraid you might be hurt in your apartment building or house?	13	5

Safety total	45	17

Social need		

Would you like help with shelter or housing?	40	15
Would you like help with obtaining food?	34	13
Would you like help with transportation?	29	11
Would you like help paying for your utility bills?	45	17
Would you like help regarding your personal or neighborhood safety?	29	11

* Answer options included the following: “doesn’t apply,” “never,” “sometimes,” “often,” “always”; positive answers included “sometimes,” “often,”, and “always.”

**Table 3:** Overlap and association of social risks and social needs.*

	Social risk, x	Overlapping social risks and social needs (xy)	Social need, y
Housing	61	32	40
Food	45	21	34
Transportation	24	16	29
Utility	11	6	45
Safety	45	18	29

* All associations between social need/risk in each domain were statistically significant with p <0.01.

**Table 4:** Association of demographic variables with social risk and social need.

	Social risk			Social need		
	
	No	Yes†	P-value	No	Yes†	P-value
Respondent			0.55			0.83
Patient	149 (88)	91 (91)		163 (89)	75 (90)	
Guardian	20 (12)	9 (9)		21 (11)	8 (10)	
Language			0.83			0.005
English	153 (91)	92 (92)		174 (95)	69 (83)	
Spanish	16 (9)	8 (8)		10 (5)	14 (17)	
Race/ethnicity			0.41			0.003
Non-Hispanic White	100 (59)	55 (55)		115 (63)	39 (47)	
Non-Hispanic Black	13 (8)	14 (14)		11 (6)	16 (19)	
Other	17 (10)	8 (8)		19 (10)	5 (6)	
Hispanic	39 (23)	23 (23)		39 (21)	23 (28)	
Gender			0.57			0.86
Male	85 (50)	57 (57)		98 (53)	43 (52)	
Female	83 (49)	43 (43)		85 (46)	40 (48)	
Other	1 (1)	0 (0)		1 (1)	0	
Insurance			0.10			< 0.001
State/public	58 (34)	50 (50)		50 (27)	58 (70)	
Private	84 (50)	38 (38)		104 (57)	16 (19)	
Self-pay/none	9 (5)	4 (4)		11 (6)	2 (2)	
Unknown	18 (11)	8 (8)		19 (10)	7 (8)	
Education			0.01			< 0.001
< 8th grade	10 (6)	12 (12)		10 (5)	12 (14)	
High School	28 (17)	29 (29)		30 (16)	27 (33)	
Some college/finished college/graduate degree	131 (77)	59 (59)		144 (78)	44 (53)	
Health literacy*			0.50			0.11
Extremely/quite a bit	144 (85)	82 (82)		159 (86)	65 (78)	
Somewhat/a little bit/not at all	25 (15)	18 (18)		25 (14)	18 (22)	

* As assessed with the question, ”How confident are you filling out medical forms by yourself?”

† “Yes” corresponds to screening positive for at least one social risk or need.

**Table 5:** Multivariable logistic regression models assessing associations between social risk and demographic variables (n = 100).

	Model 1A OR (95% CI)	Model 1B OR (95% CI)
Gender		
Male	1.00	1.00
Female	0.82 (0.49–1.39)	0.74 (0.44–1.24)
Race/ethnicity		
Non-Hispanic White	1.00	1.00
Non-Hispanic Black	1.78 (0.75–4.20)	1.81 (0.78–4.21)
Other	1.04 (0.41–2.63)	0.96 (0.38–2.43)
Hispanic	1.14 (0.53–2.45)	1.19 (0.56–2.51)
Language		
English	1.00	1.00
Spanish	0.49 (0.16–1.52)	0.65 (0.21–1.96)
Education		
< 8th grade or high school	1.00	--
Some college/finished college/graduate degree	0.44 (0.24–0.80)	--
Health literacy		
Extremely/quite a bit	--	1.00
Somewhat/a little bit/not at all	--	1.13 (0.56–2.29)
Insurance		
State/public	1.00	1.00
Private	0.61 (0.34–1.09)	0.50 (0.29–0.88)
Self-pay/none	0.55 (0.15–2.01)	0.50 (0.14–1.75)
Unknown	0.52 (0.20–1.34)	0.51 (0.20–1.29)

*OR*, odds ratio; *CI*, confidence interval.

**Table 6:** Multivariable logistic regression models assessing associations between social need and demographic variables (n=83).

	Model 2A OR (95% CI)	Model 2B OR (95% CI)
Gender		
Male	1.00	1.00
Female	1.04 (0.56–1.91)	0.97 (0.53–1.77)
Race/ethnicity		
Non-Hispanic White	1.00	1.00
Non-Hispanic Black	4.96 (1.88–13.11)	5.00 (1.91–13.12)
Other	1.31 (0.41–4.17)	1.20 (0.37–3.86)
Hispanic	0.82 (0.32–2.05)	0.88 (0.35–2.16)
Language		
English	1.00	1.00
Spanish	3.57 (1.01–12.57)	4.07 (1.17–14.10)
Education		
< 8th grade or high school	1.00	--
Some college/finished college/graduate degree	0.52 (0.27–1.02)	--
Health literacy		
Extremely/quite a bit	--	1.00
Somewhat/a little bit/not at all	--	1.32 (0.60–2.94)
Insurance		
State/public	1.00	1.00
Private	0.15 (0.07–0.30)	0.13 (0.07–0.26)
Self-pay/none	0.11 (0.02–0.59)	0.10 (0.02–0.53)
Unknown	0.33 (0.12–0.90)	0.34 (0.13–0.92)

*OR*, odds ratio; *CI*, confidence interval.

## References

[1] Center for Health Care Strategies (2017). Screening for Social Determinants of Health in Populations with Complex Needs: Implementation Considerations.

[2] World Health Organization About social determinants of health.

[3] Chetty R, Stepner M, Abraham S (2016). The association between income and life expectancy in the United States, 2001–2014. JAMA.

[4] Kushel MB, Gupta R, Gee L (2006). Housing instability and food insecurity as barriers to health care among low-income Americans. J Gen Intern Med.

[5] Beck AF, Cohen AJ, Colvin JD (2018). Perspectives from the Society for Pediatric Research: interventions targeting social needs in pediatric clinical care. Pediatr Res.

[6] Gottlieb LM, Hessler D, Long D (2016). Effects of social needs screening and in-person service navigation on child health: a randomized clinical trial. JAMA Pediatr.

[7] Berkowitz SA, Hulberg AC, Hong C (2016). Addressing basic resource needs to improve primary care quality: a community collaboration programme. BMJ Qual Saf.

[8] Wiley Online Library (2018). Material needs of emergency department patients: a systematic review - Malecha - 2018.

[9] Rodriguez RM, Fortman J, Chee C (2009). Food, shelter and safety needs motivating homeless persons’ visits to an urban emergency department. Ann Emerg Med.

[10] Tang N, Stein J, Hsia RY, Maselli JH, Gonzales R (2010). Trends and characteristics of US emergency department visits, 1997–2007. JAMA.

[11] Alderwick H, Gottlieb LM (2019). Meanings and misunderstandings: a social determinants of health lexicon for health care systems. Milbank Q.

[12] Bottino CJ, Rhodes ET, Kreatsoulas C (2017). Food insecurity screening in pediatric primary care: Can offering referrals help identify families in need?. Acad Pediat.

[13] Losonczy LI, Hsieh D, Wang M (2017). The Highland Health Advocates: a preliminary evaluation of a novel programme addressing the social needs of emergency department patients. Emerg Med J.

[14] Billioux A, Verlander K, Centers for Medicare and Medicaid Services (2017). Standardized screening for health-related social needs in clinical settings: the Accountable Health Communities Screening Tool. NAM Perspectives.

[15] Garg A, Sheldrick RC, Dworkin PH (2018). The inherent fallibility of validated screening tools for social determinants of health. Acad Pediat.

[16] Henrikson NB, Blasi PR, Dorsey CN (2019). Psychometric and pragmatic properties of social risk screening tools: a systematic review. Am J Prev Med.

[17] Breese PE, Burman WJ, Goldberg S (2007). Education level, primary language, and comprehension of the informed consent process. J Empir Res Hum Res Ethics.

[18] Montez JK, Hummer RA, Hayward MD (2012). Educational attainment and adult mortality in the United States: a systematic analysis of functional form. Demography.

[19] Gottlieb L, Hessler D, Long D (2014). A randomized trial on screening for social determinants of health: the iScreen study. Pediatrics.

[20] Anderson ES, Lippert S, Newberry J (2016). Addressing social determinants of health from the emergency department through social emergency medicine. West J Emerg Med.

[21] Doran KM, Misa EJ, Shah NR (2013). Housing as health care--New York’s boundary-crossing experiment. N Engl J Med.

[22] James TL, Bibi S, Langlois BK (2014). Boston Violence Intervention Advocacy Program: a qualitative study of client experiences and perceived effect. Acad Emerg Med.

[23] Basu S, Berkowitz SA, Seligman H (2017). The monthly cycle of hypoglycemia: an observational claims-based study of emergency room visits, hospital admissions, and costs in a commercially insured population. Med Care.

[24] Centers for Disease Control and Prevention (2016). National Hospital Ambulatory Medical Care Survey: 2016. Emergency Department Summary Tables.

[25] Martel ML, Klein LR, Hager KA (2018). Emergency Department Experience with Novel Electronic Medical Record Order for Referral to Food Resources. West J Emerg Med.

[26] Baggett TP, Singer DE, Rao SR (2011). Food insufficiency and health services utilization in a national sample of homeless adults. J Gen Intern Med.

[27] Manchanda R, Gottlieb L (2015). Research on Integrating Social & Medical Care | SIREN | HealthBegins Upstream Risk Screening Tool.

[28] (2018). Health Leads. The Health Leads Screening Toolkit.

[29] Food Research & Action Center Addressing food insecurity: a toolkit for pediatricians.

